# Continuity of the Middle Stone Age into the Holocene

**DOI:** 10.1038/s41598-020-79418-4

**Published:** 2021-01-11

**Authors:** Eleanor M. L. Scerri, Khady Niang, Ian Candy, James Blinkhorn, William Mills, Jacopo N. Cerasoni, Mark D. Bateman, Alison Crowther, Huw S. Groucutt

**Affiliations:** 1 Pan-African Evolution Research Group, Max Planck Institute for the Science of Human History, Kahlaische Straße 10, 07745 Jena, Germany; 2 Department of Classics and Archaeology, University of Malta, Msida, Malta; 3 Institute of Prehistoric Archaeology, University of Cologne, 50931 Cologne, Germany; 4 Département D’Histoire, Université Cheikh Anta Diop de Dakar, BP. 5005, Dakar-Fann, Senegal; 5 Centre for Quaternary Research, Department of Geography, Royal Holloway, University of London, London, UK; 6 School of Archaeology, University of Oxford, 1 South Parks Road, Oxford, OX1 3TG UK; 7 Department of Geography, Winter St., University of Sheffield, Sheffield, S10 2TN UK; 8 School of Social Science, The University of Queensland, St Lucia, 4072 Australia; 9 Department of Archaeology, Max Planck Institute for the Science of Human History, Kahlaische Straße 10, 07745 Jena, Germany; 10 Extreme Events Research Group, Max Planck Institutes for Chemical Ecology, the Science of Human History, and Biogeochemistry, 07745 Jena, Germany

**Keywords:** Archaeology, Cultural evolution

## Abstract

The African Middle Stone Age (MSA, typically considered to span ca. 300–30 thousand years ago [ka]), represents our species’ first and longest lasting cultural phase. Although the MSA to Later Stone Age (LSA) transition is known to have had a degree of spatial and temporal variability, recent studies have implied that in some regions, the MSA persisted well beyond 30 ka. Here we report two new sites in Senegal that date the end of the MSA to around 11 ka, the youngest yet documented MSA in Africa. This shows that this cultural phase persisted into the Holocene. These results highlight significant spatial and temporal cultural variability in the African Late Pleistocene, consistent with genomic and palaeoanthropological hypotheses that significant, long-standing inter-group cultural differences shaped the later stages of human evolution in Africa.

## Introduction

The African Middle Stone Age (MSA) is a cultural phase characterized by features such as a focus on prepared core lithic technology, hafting, and long-distance exchange, that emerged synchronously with the biological appearance of our species, *Homo sapiens*^1–5^ (see Supplementary Materials [SM]). Together with these characteristics, the spatial and temporal distribution of the MSA across Africa between ca. 300–30 ka is seen as being relatively homogenous, and the term has also been used as a chronological marker (e.g.,^6^). While behavioural and cultural complexity is increasingly recognized in the MSA (e.g.,^1–5^), the transition to the Later Stone Age (LSA), with features such as miniaturized lithic technology and ostrich eggshell beads, is often seen as a seminal turning point in human history and the establishment of the first societies analogous to those characterizing recent humans^7–9^. To some, the transition was so dramatic as to suggest it was caused by a cognitive mutation that marks the appearance of truly ‘modern humans’^9,10^.

Recent research across Africa challenges this view of a simple, abrupt, continent-wide, transition from the MSA to the LSA (SM). The transition is gradual at some sites^11^, and begins as early as ca. 67 ka in some cases^12^. At other sites the transition occurs much later^11,13^, with late MSA assemblages often characterised by the same classic features documented in early MSA assemblages^2,3,14^. Growing evidence that human biological and cultural evolution was a Pan-African process^15,16^ has also crystallized the regional spatial and temporal dynamics of the MSA and LSA as a key factor for understanding our species evolutionary history. In a continent where preserved Pleistocene biological remains are rare, material culture offers a rich record of human behaviour, which is both significant in itself and a crucial parameter in biologically focused models of human evolution.

Paleoanthropological research in West Africa highlights the distinct character of this poorly understood region. Although the only known Pleistocene *Homo sapiens* fossil from the area comes from an LSA context in Nigeria dating to ca. 16–12 ka, the Ihò Eleru (previously incorrectly labelled ‘Iwo Eleru’) calvaria displays morphological features typically found in much earlier human populations^17,18^. This shows that material culture 'stages' and skeletal morphology are not necessarily coupled, and critically, that there may have been a regional survival of a distinct and perhaps relatively isolated population until the end of the Pleistocene^19^. Likewise, genetic analyses highlight West Africa as a key wellspring of our species' genetic diversity^19^, with some studies even suggesting contributions from past African archaic populations^20^.

The archaeological record also indicates a distinctive character to human prehistory in this area. In West Africa, the argument for a young Middle Stone Age (MSA) has been made since at least the 1970s, typically on the basis of geomorphology and early radiocarbon dating (see^21–24^ for summaries). Early studies of sites from across the region including those in Ghana^23^, Senegal^24^ and Niger^25^ led to the prediction that the MSA in West Africa should date to between 35 and 15 ka^26^, as indeed optically stimulated luminescence (OSL) dating at Birimi in Ghana demonstrated^26^. More recently, work on various localities at Ounjougou and in the Lower Falémé valley have identified lithic assemblages from Marine Isotope Stage (MIS) 6 to MIS 2 which feature varied technologies within an MSA umbrella (e.g.^22,27–29^). This work is further elaborated by work at Tiémassas, coastal Senegal, where three classic MSA assemblages span 62–25 ka^30,31^ and in the Lower Senegal Valley^32,33^ where MSA assemblages have been dated to 12 ka. These sites share a consistent focus on Levallois reduction approaches, sometimes complemented by discoidal methods, alongside the appearance of tools such as scrapers, denticulates and retouched points, which characterise the West African MSA. Critically, these assemblages lack technological features attributed to the earliest LSA assemblages in the region, including bipolar and blade reduction and backing, which do not appear until ca. 16–12 ka^17,21,22,27,34^. However, the majority of West African MSA sites offer limited chronological control (e.g.^23–25^), demanding clear demonstrations of the young timeframe of their occurrence to successfully integrate them into broader evolutionary models.

## Results

**Figure 1:**
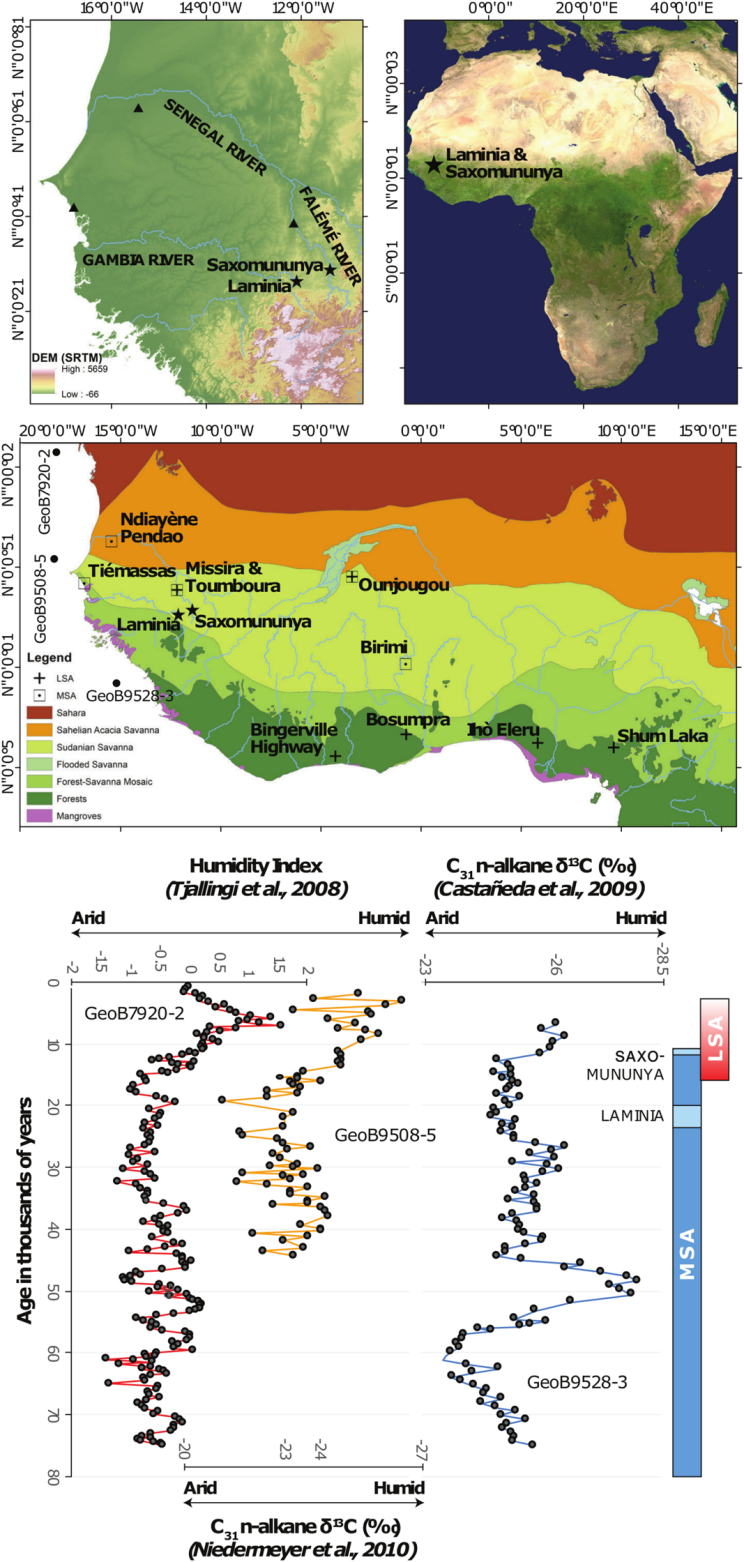
Site locations and chronological details. Maps illustrating the location of Laminia and Saxomununya in relation to the topography and major fluvial systems of Senegal (top left; SRTM DEM^63^), their position within Africa (top right; Image: NASA), and in respect to other key dated West African MSA and LSA sites, emphasizing the association of the MSA sites with Sudanian savannahs and the early LSA sites with tropical forest ecologies (centre yy^64^). (Bottom) The chronology of MSA (blue) and LSA (red) with the ages of Laminia and Saxomununya highlighted in light blue, against three Marine Core datasets indicating substantial directional change in humidity (left; *48*) and patterns of vegetation (centre (*49*); right (*50*)) at the MSA-LSA transition.

Here we report late MSA assemblages from the sites of Laminia and Saxomununya, Senegal (Fig. 1), dating to ca. 22–21 ka and less than ca. 11 ka, respectively. These dates confirm the continuity of MSA technologies in West Africa thousands of years after they had been replaced by the LSA elsewhere on the continent. Both sites consist of river sediments that comprise the lowest terrace unit within the reach of each of their respective catchments; the Gambia River in the case of Laminia, and the Falémé river in the case of Saxomununya. The low rate of tectonic uplift that is found in this region means that unlike many large river systems, neither the Gambia nor the Falémé river valleys contain extensive sequences of terrace units. This observation is supported by the study of digital elevation models (DEMs) of the study sites, which show limited topographic evidence for multiple discrete terrace surfaces (Figs. S1; S2, see SM). Instead, in the study reaches, the near channel geomorphology consists of a single terrace surface, 5–7 m above the modern channel which is entrenched by gullies and tributary channels. The studied sediments at Laminia are found within exposures of this terrace feature whilst the site of Saxomununya is found on the terrace surface.

**Figure 2:**
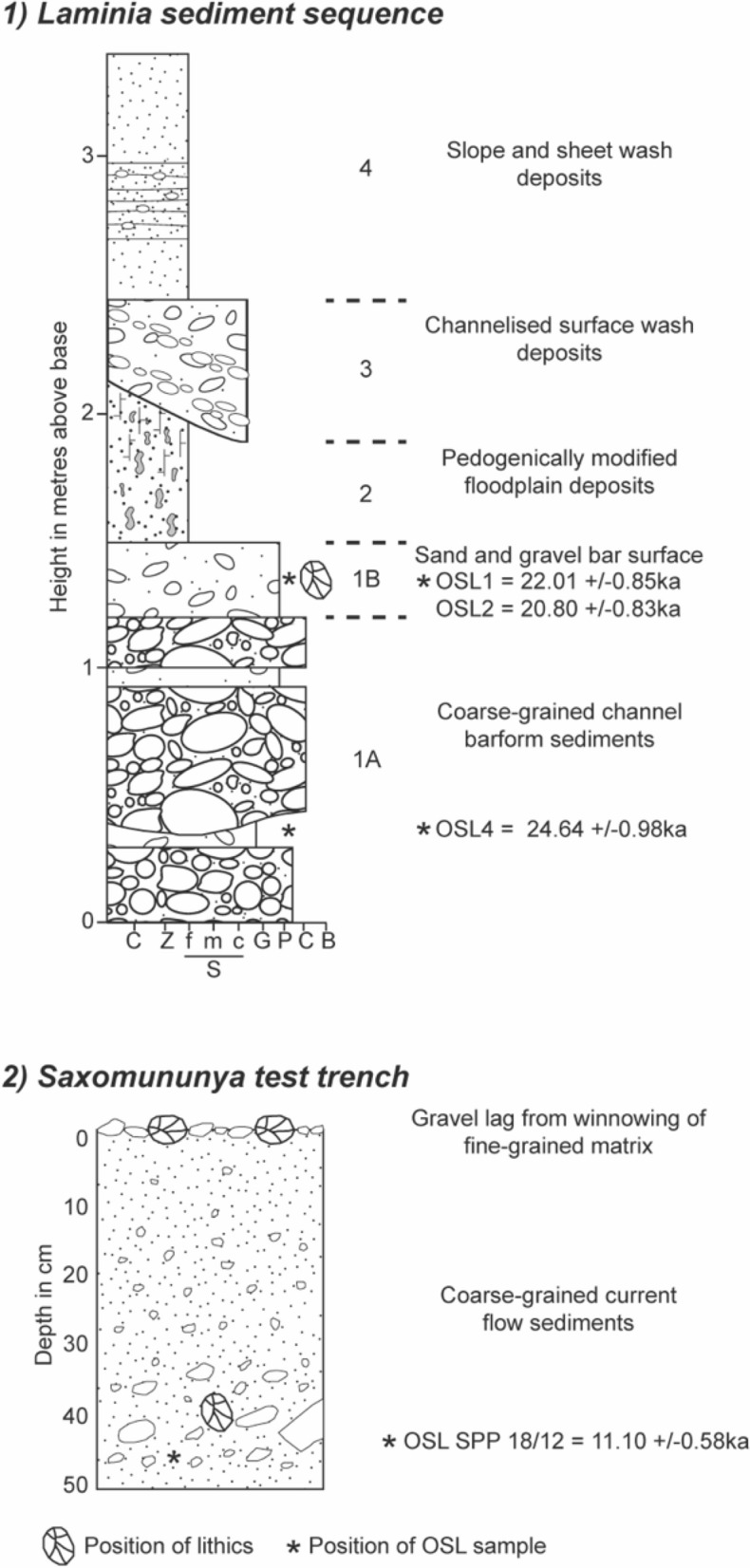
Sedimentary sequences from Laminia (1) and Saxomununya (2).

The site of Laminia is exposed in sections of a south bank terrace of the Gambia river. These exposures, approximately 3 m in height, consist of a lower unit of cobble/pebble dominated well-sorted gravels overlain by fine-grained sands and silts (Fig. 2, Figs. S3, S4, see SM). The sequence is interpreted as reflecting the downstream migration of a large bar-form under high flow conditions in the main channel followed by lower energy deposition, most likely in the overbank environment after the river has undergone lateral migration. The lithic artefacts at Laminia come from a very specific context, the upper 0.2 m of the gravel deposits (Unit 1B). No other units have yielded lithic artefacts. In the context of the geomorphology of the site the position of the lithic finds is key because they occur on top of the bar-form. The absence of these lithics from elsewhere within the gravels implies that the artefacts were not being reworked downstream as part of the bedload but are concentrated at the bar surface and accumulated after the bar had stabilized. We therefore interpret the presence of artefacts here as relating to exploitation of the stabilized bar as a raw material resource, the stone tools which were produced on site are therefore in situ.

The site of Saxomununya occurs on a river terrace surface on the west side of the Falémé river (Fig. 2, Figs. S5, S6, see SM). Much of the site consists of exposed bedrock, but to the south there is a shallow accumulation of sediments (SM). A 0.5-m deep trench was excavated into the terrace to examine the nature of the sediments that make up the land surface. The shallow sediments underlying the land surface consist of well-sorted coarse sands and pebbles with occasional cobbles. Generally, these deposits are structureless except for some weakly developed cross-bedding. The sediments are typical of deposition in an active fluvial environment. The terrace surface is rich in lithic artefacts, whilst five buried artefacts were recovered from excavation. The freshness, density, and size range of the artefacts implies that whilst the sediments are fluvial in origin the lithic assemblage itself is in situ, and has been left there by humans following the cessation of fluvial activity (SM). We interpret Saxomununya as a raw material source, where humans used the gravel deposit to produce stone tools.

**Figure 3:**
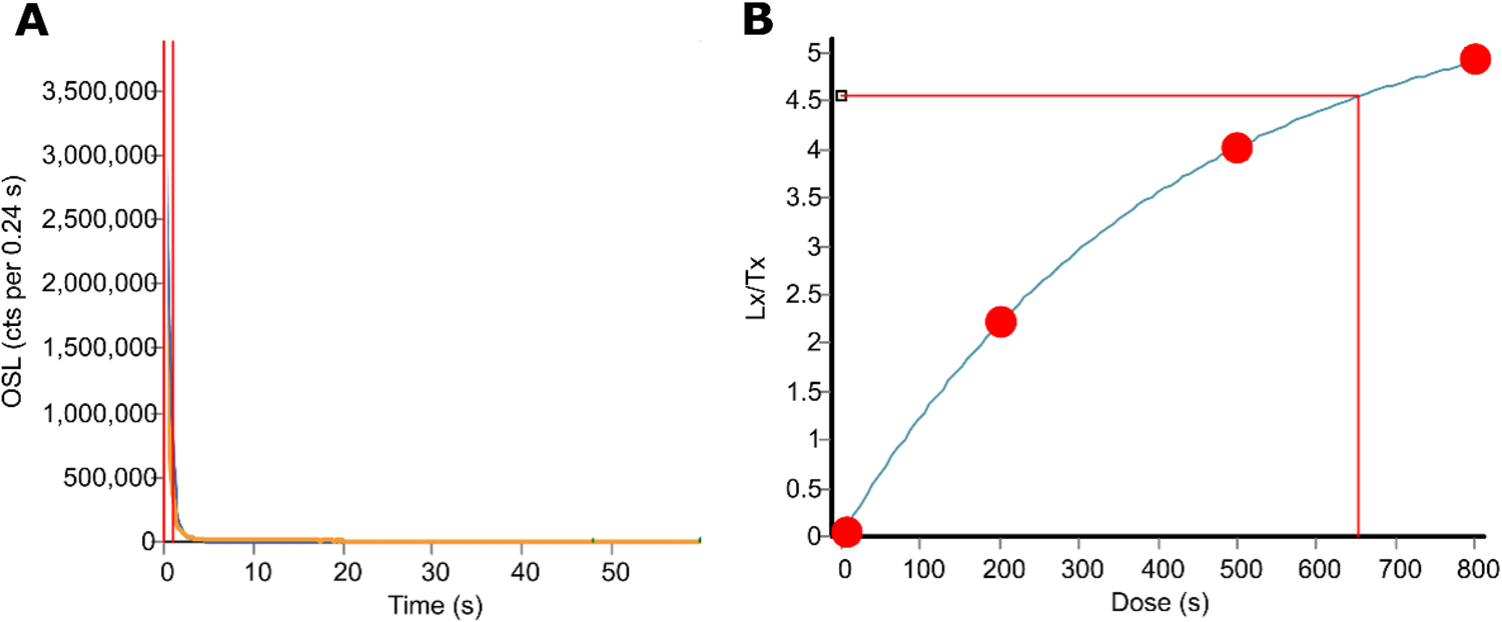
Examples of OSL measurements from Shfd16117. (**A**) A shine down curve showing rapid decay of the OSL signal with stimulation indicative of a signal dominated by the fast component. (**B**) A Single Aliquot Regenerative (SAR) growth curve showing low thermal transfer as the zero point is close to zero and good fit of growth to laboratory doses.

**Figure 4:**
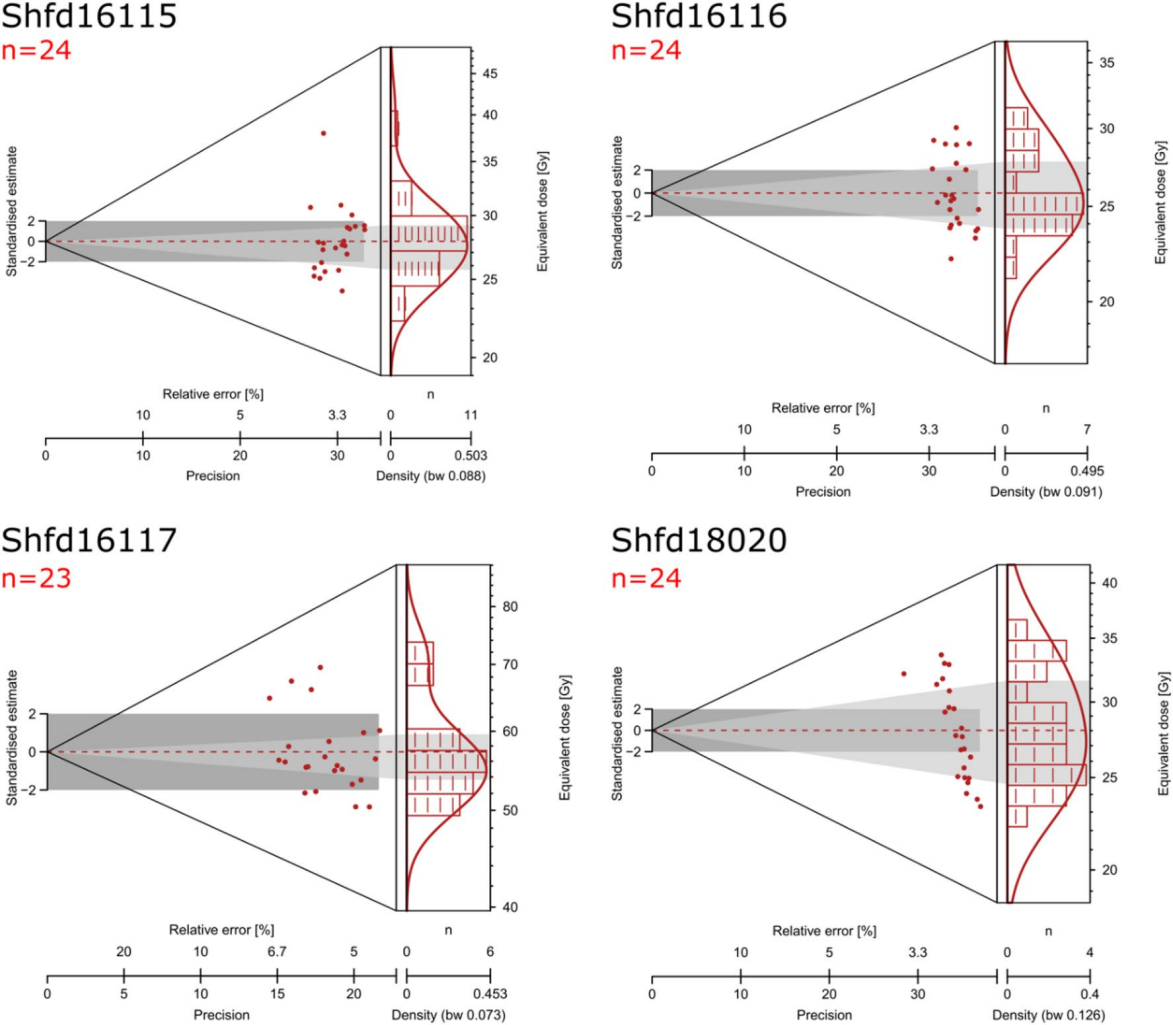
Abianco plots of OSL palaeodoses (De). These are shown for the three samples from Laminia (Shfd16115-17) and the sample from Saxomununya (Shfd18020).

We used optically OSL dating of quartz grains to date Laminia and Saxomununya. All samples were found to have good OSL characteristics, with fast OSL signal depletion with stimulation and high De replicate reproducibility taken to indicate good signal resetting prior to burial (Figs. 3, 4, Tables 1, 2). Three OSL samples were taken at Laminia (Fig. 2.1, Shfd16115-17, SM) which produced consistent ages of 24.6 ± 0.98 ka for Unit 1A and 22.0 ± 0.85 ka and 20.8 ± 0.83 ka Unit 1B. At Saxomununya, we dated the terrace deposits that were used as a raw material source to offer a *terminus post quem* of the human occupation. The OSL sample was taken from the base of the trench, which sits just above bedrock. The sample (Shfd18020) produced an age of 11.1 ± 0.58 ka. These chronometric age estimates are consistent with geomorphological interpretations of the sites, namely that they both represent the most recent stage of terrace formation.

**Table 1:** Summary of results for dosimetry data. ^+^ Cosmic dose is calculated as a linear decay curve at depths below 50 cm. Above this depth, errors in calculation may lead to an under-estimation of the cosmic dose contribution. * Total Dose is attenuated for grain size, density and moisture.

Lab code	U (PPM)	Th (PPM)	Rb (PPM)	K (%)	D_cosmic_^+^ (μGy/a^−1^)	Moisture (%)	Dose rate* (μGy/a^−1^)
Shfd16115	1.60	5.1	38.9	0.5	123 ± 6	3	1247 ± 44
Shfd16116	1.60	5.1	38.9	0.5	123 ± 6	3	1240 ± 43
Shfd16117	3.71	9.7	48.9	0.6	138 ± 7	5	2219 ± 82
Shfd18020	6.19	7.8	20	0.3	189 ± 9	4	2527 ± 112

**Table 2:** Summary of OSL results from Laminia (Shfd16115-17) and Saxomununya (Shfd18020), presented with one sigma confidence intervals which incorporate systematic uncertainties with the dosimetry data, uncertainties with the palaeomoisture content and errors associated with the De determination.

Lab Code	Field Ref	Depth (cm)	De (Gy)	OD (%)^a^	Dose rate (μGy/a^−1^)	Age (ka)
Shfd16115	OSL 1	370	27.44 ± 0.30	10	1247 ± 44	22.0 ± 0.85
Shfd16116	OSL 2	370	25.79 ± 0.50	9	1240 ± 43	20.8 ± 0.83
Shfd16117	OSL 4	280	54.68 ± 0.78	10	2219 ± 82	24.6 ± 0.98
Shfd18020	SPP18/12	45	28.00 ± 0.74	13	2527 ± 112	11.1 ± 0.58

^a^ Overdispersation of De data.

**Figure 5:**
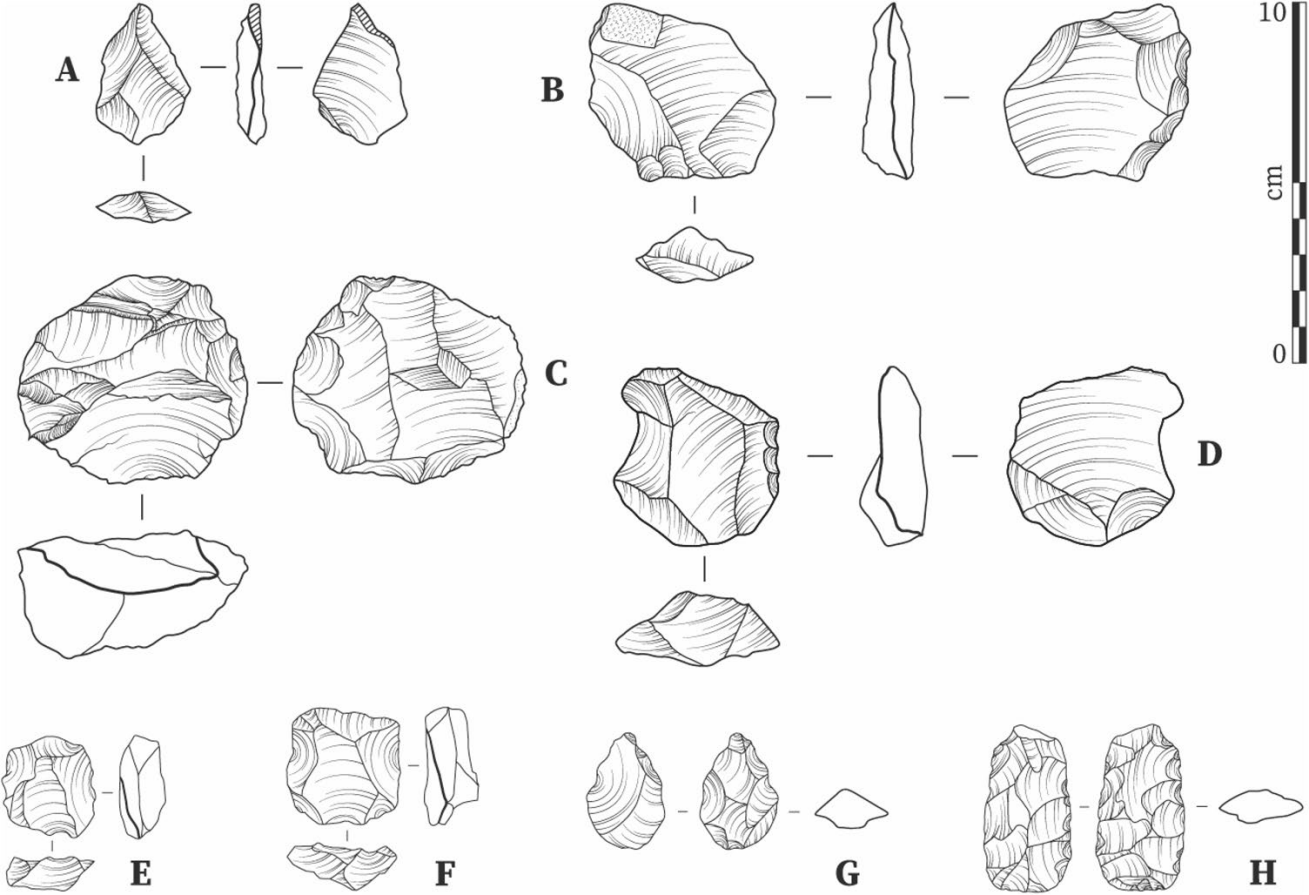
Lithics from Laminia (**A–D**) and Saxomununya (**E–H**)**.** (**A**) unretouched flake; (**B**) bifacially retouched flake; (**C**) Levallois core evidencing a step fracture; (**D**) side retouched flake/scraper; (**E**, **F**) Levallois cores; (**G**) bifacial foliate point; (**H**) bifacial foliate. Figure licensed under CC-BY-4.0.

Surprisingly, given the young ages of these sites, both Laminia and Saxomununya feature classic MSA technological characteristics. At Laminia, 30 lithics in a fresh condition were recovered directly from the upper part of Unit 1B (Figs. 2, 5, S4, S7) while a further 85 were found in the immediate vicinity in the downstream direction alongside evidence for the localized erosion of Unit 1B. Complete artefacts comprise 48 cores and 56 flakes (SM). The assemblage primarily consists of artefacts made from small cobbles/large pebbles of quartz, with only three cores made on quartzite, and displays a homogeneous technological character. Nodules with natural Levallois-like convexities were typically selected for flaking. Of the 48 cores, early stage cores, defined by their large size and the presence of few scars with high levels of cortex, are varied in their typology, as might be expected. This typology includes single platform (n = 7), multiplatform cores (n = 6) and tested pebbles (n = 5). These cores exhibit a high number of aberrant terminations (38%), and many appear to have flaws in them, such as natural plains, which make knapping difficult. Four bidirectional cores seem to represent a slightly more advanced phase of reduction. Aside from two broken cores (one Levallois) the remaining 24 cores—making up 50% of the total number of cores – are Levallois cores, centripetally prepared for preferential removals. Where core reduction extends beyond preliminary nodule testing, Levallois reduction is clearly dominant.

The abundance of cores suggests that the site was targeted for raw material extraction, although removal of finer debitage from the site cannot be discounted. However, a notable proportion of the flakes (n = 44; ca. 66%) preserve cortex, extending over half the dorsal surface in 18% (n = 12) of cases, supporting the suggestion for a focus on primary reduction activities. The character of the debitage assemblage is consistent with the core technology, comprising facetted Levallois flakes alongside preparatory and débordant flakes, indicating that Levallois reduction schemes are the dominant technological feature of the assemblage. Retouched tools are rare, further supporting the focus on primary reduction activities at the site. The focus on preferential Levallois reduction, with centripetal preparation evident in both core and debitage assemblage is entirely consistent with an MSA attribution (Fig. 5, Fig. S7).

At Saxomununya, the lithic assemblage was collected in a spatially limited area of ca. 20 by 30 m, beyond which artefacts were very sparse. The total collected and excavated assemblage consisted of 231 cores, 336 flakes, 29 retouched flakes and 87 pieces of debitage and shatter. The predominant raw material consists of small quartz pebbles, while a few pieces of quartzite in the form of flakes are present. The small package size of the available raw material is reflected in the size of the lithics. These are small, but clearly not decoupled from raw material package size, a fact also reflected by the fact that no reduction techniques associated with the specific production of miniature lithics was observed. All the cores and retouched flakes, and just over half the flakes (192, randomly selected) were studied in detail (SM). Many of the cores (61 or 26.4%) are classic preferential Levallois cores with centripetal preparation. The three excavated cores belong to this category. A further 14 cores (6.1%) are Levallois core fragments or centripetally prepared but unstruck Levallois cores. Many of the 77 (33%) multiplatform or amorphous cores also display some evidence of previous Levallois-like exploitation (e.g. remnants of a central plane), suggesting discard once Levallois reduction schemes were no longer possible. A further 26 single platform cores (11.3%) display some evidence of core hierarchy, with the natural convexities of the pebbles used to detach relatively large flakes. Finally, eight cores (3.5%) are discoidal with a further 18 cores (7.8%) displaying a pattern of radial flaking but without evidence for platform preparation evident amongst the Levallois core sample.

The debitage is predominately comprised of small (mean = 32 mm length) complete flakes. Fifteen (7.8%) Levallois flakes with finely facetted platforms are present alongside eight (4.2%) débordant flakes, which maintain flaking surface convexities in Levallois reduction systems. A single pyramidal flake preserving an entire discoidal flake surface is present. The difficulty in knapping is well demonstrated by frequent aberrant terminations on flakes and cores (overshot flakes, step terminations) and siret fractures on the flakes. Twenty-nine flakes are retouched into classic MSA tool types, including denticulates, side and end scrapers, notches and retouched Levallois flakes that are well documented in MSA assemblages in West Africa and peripheral regions (e.g.,^2,21^). Three small foliate fragments, including two broken tips, were also recovered, as well as one whole foliate (Fig. 5, Fig. S8) and three possible foliate preforms. These tools resemble other Late Pleistocene examples recorded in the Lower Falémé. Although these are some distance away from Saxomununya, they are assemblages described as 'MSA-like', also featuring side-scrapers^28^. Overall, the core, debitage and tool assemblage, characterised by the presence of Levallois and discoidal reduction schemes and the absence of bipolar or laminar reduction, can best be ascribed to the MSA (S2, Fig. 5, Fig. S8), with the small size of artefact attributable to the limitations of available raw materials.

## Discussion

The dates of ca. 11 ka from Saxonomunya and ca. 21–24 ka from Laminia sit at the end of a chronological arc of MSA assemblages in West Africa. These include ca. 11.6 ka at Ndiayène Pendao^33^, ca. 20–50 ka at Birimi^26^, ca. 33 ka at Toumboura III^28^, and ca. 25–62 ka at Tiémassas^30,31^. This sequence demonstrates that MSA technologies persisted, rather than being reinvented, in the terminal Pleistocene. Our results mean that young MSA assemblages are now known from all of the major fluvial systems in the area; Laminia from the Gambia, Saxonomunya from the Falémé, Ndiayène Pendao from the Senegal, and Tiémassass from the Saloum. Although they represent the final phase of the MSA in the region, neither Laminia nor Saxonomunya yield any elements characteristic of the LSA. They are classically MSA in their composition, rather than indicative of a transitional phase between the MSA and LSA.

The record of a young MSA in West Africa builds on, consolidates and extends previously reported hints of a young MSA elsewhere in Africa, which have often been viewed as isolated or exceptional phenomena (e.g.^35,36^). Along with recent studies such as those as those extending the chronology of the LSA back in time (e.g.^12^), the young MSA of West Africa therefore adds to the evidence that the MSA to the LSA transition was highly variable in both chronology and character. West Africa presents particularly persuasive evidence of a late MSA: these assemblages comprise a suite of classic MSA characteristics that are absent in the earliest LSA in the region, which appears late with a discrete combination of stone tool technologies. This situation contrasts with some other regions of Africa, such as East Africa. Elements of stone tool technologies that are dominant features of LSA assemblages, such as the use of backing, blade production and bipolar reduction methods, are clearly apparent in MSA assemblages from MIS 5^37^, whereas some ‘MSA-like’ elements may be reinvented in the Holocene^38^. At Panga ya Saidi in coastal Kenya, a major transition occurs from a classic late MIS 5 MSA to assemblages from ca. 67 ka characterized by small size and a focus on fine grained raw material^12^. Within the latter, various technologies (bipolar, blade etc.) alternate in frequency. Likewise, Levallois technologies re-appear. Such technological overlaps do not prohibit differentiation of the MSA and LSA in eastern Africa (e.g.^39^), but stand in stark contrast to West African records where late MSA assemblages lacking LSA-features clearly persist.

An interesting corollary to the demonstration of a young MSA in West Africa is the late onset of the LSA in the same region. LSA assemblages are present in the western part of Central Africa by ca. 30 ka^40^. LSA assemblages first appear in West Africa (i.e. west of Cameroon) ca. 16–12 ka in the forested regions of the modern countries of Nigeria, Côte d’Ivoire, and Ghana^17,18,21,22^. The LSA then appears further west and north in the Falémé Valley from around 11 ka^22^. The earliest LSA assemblages lack MSA features and emphasize the production of geometric microliths on small laminar blanks. The LSA does not seem to occur in West Africa until shortly before the use of ceramics and then the development of agriculture^41^. While much work remains to be carried out exploring the nuances of the spatial and temporal patterning in the transition from the MSA to the LSA, West Africa appears to follow an environmental dynamic, at least in some regards. The appearance of the LSA in the forested region of eastern and central West Africa correlates with an expansion of forests in the Terminal Pleistocene, around 15 ka^42–47^. On the basis of current data, transitions between glacial and interglacial peaks were unlikely to be smooth, leading to the formation of ecological bottlenecks that were non-synchronous between species^45^. Niang and colleagues^30^ have suggested that occupation of ecotonal habitats, such as at Tiémassas where the MSA site is located in close proximity to Sudanian savannahs, Guinean mixed forest-savannahs and mangroves, may have played a role in fostering engagement with new ecological settings. Finally, time transgressive peaks in humidity also shed some light on the persistence of the MSA in the low latitudes and related temporally and spatially patchy cultural turnovers^42^.

These findings suggest that a profound cultural turnover occurred in West Africa around the transition from the Pleistocene to the Holocene. In Senegal, at the westernmost point of the entire mainland Old World, the youngest known MSA at ca. 11 ka and the oldest LSA at ca. 11 ka occur within the same valley, with no technological overlap. This suggests the existence of strong cultural divisions in the Terminal Pleistocene and early Holocene of West Africa. The extent to which this division was biological versus cultural remains to be elucidated; but at a broad scale the end result is the same, that panmixia (random mating) is unlikely and that strong population subdivisions were present. The end of the MSA in West Africa occurs around a time of increasing humidity and forest growth (Fig. 1;^48–50^). This provides context for the spread of the LSA into areas which may have been relatively isolated by ecological barriers and bottlenecks^47^ (SM). The relatively sudden increase in humidity from around 15 ka matches the age of the appearance of the LSA in West Africa, with the subsequent transition to the peak conditions of the African Humid Period from around 11 ka correlating with the end of the MSA and the spread of the LSA into Senegal^42^.

Our results, along with other recent findings across Africa, demonstrate that the transition from the MSA to LSA is a spatially and chronologically heterogenous process of behavioural change. These findings do not fit a simple unilinear model of cultural change towards ‘modernity’, nor the use of these terms as specific, continent-wide chronological markers. Indeed, the continuity of a fairly generic MSA into the Holocene adds to a body of evidence showing that spatial and temporal expressions of complexity in the MSA are patchy (e.g.*,*^2^), indicating that motivations to invest in technological innovation related to factors other than simple behavioural capacity (e.g. ^14^.). Groups of hunter-gatherers embedded in radically different technological traditions occupied neighbouring, and sometimes perhaps shared, regions of Africa for thousands of years. This is at least consistent with the genetic signal for strong population structure seen in the last ca. 15 thousand years^18,51^. Elucidating the diverse relationships between populations is crucial for understanding the evolution of our species and offers an alternative to the extrapolation of the record from individual sites/regions to a continental scale.

## Materials and methods

### Fieldwork

Fieldwork in southeastern Senegal was undertaken between 2016 and 2018, as part of the Senegal Prehistory Project, which aimed to identify the presence of Stone Age occupations and understand their contexts. Previous survey work indicated that preservation of Late Pleistocene sediment archives was likely to be limited, resulting from considerable recycling of sediments within modern valleys, and limited topographic diversity across low altitude landscape. Additionally, thick, erosion resistant ferricrete profiles largely prohibit any substantial remodeling of the landscape. As a result, our survey aimed to target the margins of the modern valleys focusing on identifying older deposits that had not yet been eroded away. The focus of the survey was on the upper part of the Falémé in Senegal, and the Senegalese section of the Gambia River and its tributaries. Previous work on the fluvial geomorphology of the region is reported by Michel^52^. Although stratified archaeological deposits were scarce, where surface and isolated finds were recovered during the course of the survey, they typically fitted with technologies that are characteristic of the Middle Stone Age (MSA) in the region. We report here two key sites from this area. Laminia (12.6425 N, 12.1075 W) is a gravel deposit on the Gambia River, and was first identified in the 1960s^53,54^. Only a small number of lithics were previously collected, and no chronometric dating had been applied to the site. Saxomununya (field code SPP18-12) is a newly discovered site in the upper part of the Falémé Valley (12.8836 N, 11.4051 W) occurring on a terrace on the west side of the river.

### OSL dating methodology

OSL samples were collected at Laminia and Saxomununya by hammering opaque metal tubes into freshly cleaned sediment sections. Three samples were collected at Laminia: Shfd16115 and Shfd16116 were located adjacent to each other at 370 cm depth from the modern surface, and Shfd16117 from 280 cm depth from the modern surface, with this variation in depth resulting from an uneven thickness of overlying, sloping deposits in Unit 4 (Fig. 2). At Saxomununya, the sample was taken from the trench at a depth of 45 cm. All samples were then transported to the Sheffield Luminescence Dating Laboratory, Department of Geography, University of Sheffield (UK) for analysis.

Quartz samples for OSL dating were prepared from the size range 125–180 μm as per Bateman and Catt^55^. OSL measurements were conducted in a Risø DA-20 luminescence reader fitted with blue/green LEDs for stimulation and signal detected was through Hoya U340 filters. Samples were mounted as a 2 mm diameter monolayer on 9.6 mm diameter aliquots with 24 replicates of palaeodoses (D_e_) measured using the single aliquot regeneration (SAR) protocol^56^. A preheat of 260 °C for 10 s was used for the Laminia site and 200 °C for 10 s for the Saxomununya site. Both were derived experimentally using dose-recovery preheat plateau tests^56^. Overall, the samples showed rapid OSL decay curves, good recycling and low thermal transfer (Fig. 4). D_e_ distributions showed the samples to be normally distributed with low overdispersion (Fig. 5, Table 2). The final derived D_e_ values were therefore based on the Central Age Model^57^. Dose rates were based on concentrations of potassium, uranium and thorium as determined by ICP-MS and ICP-OES. All dose rates were appropriately attenuated for moisture (based on present-day values) and sediment size and incorporated a cosmic dose rate following the published algorithm of Prescott & Hutton^58^.

### Lithic analysis

Lithic analysis methods follow those previously reported by Scerri and colleagues^59,60^ and Groucutt and colleagues^61,62^. We recorded detailed technological and metric features of the entire Laminia assemblage. For the larger Saxomununya assemblage, we studied cores and retouched flakes in more detail and a random sample of just over half (192) of the complete flakes.

The aim of the lithic analysis was to characterize the fundamental techo-typological features of the assemblage, as well as to assess characteristics such as weathering and breakage patterns. We therefore classified each studied lithic into raw material types, evaluated its weathering, and evaluated indications of knapping technique (e.g. percussor form), gave each piece a typological classification, and recorded technological features such as dorsal scar pattern, measured mass (grams) using digital scales, and recorded metric dimensions using digital calipers.

## Supplementary Information

Supplementary Information.
