# Supplementary material for: Continuity of the Middle Stone Age into the Holocene
Source: Sci Rep. 2021 Jan 11;11:70. doi: 10.1038/s41598-020-79418-4 (PMC7801626; doi:10.1038/s41598-020-79418-4)
Supplement: Supplementary file 1 — Supplementary Information. [file 41598_2020_79418_MOESM1_ESM.docx]

**Supplementary Materials**

**Continuity of the Middle Stone Age into the Holocene**

Eleanor M.L. Scerri^1,2,3*^, Khady Niang^4^, Ian Candy^3^, James Blinkhorn^1,5^, William Mills^6^, Jacopo N. Cerasoni^1^, Mark D. Bateman^7^, Alison Crowther^8,9^, Huw S. Groucutt^3,9,10^

^1^Pan-African Evolution Research Group, Max Planck Institute for the Science of Human History, Kahlaische Straße 10, 07745, Jena, Germany.

^2^Department of Classics and Archaeology, University of Malta, Msida, Malta

^3^ Institute of Prehistoric Archaeology, University of Cologne, Cologne, 50931, Germany.

^4^Département d’Histoire, Université Cheikh Anta Diop de Dakar, BP. 5005 Dakar-Fann, Senegal.

^5^Centre for Quaternary Research, Department of Geography, Royal Holloway, University of London, U.K.

^6^School of Archaeology, University of Oxford, 1 South Parks Road, Oxford OX1 3TG, U.K.

^7^Department of Geography, Winter St., University of Sheffield Sheffield S10 2TN, U.K.

^8^School of Social Science, The University of Queensland, St Lucia 4072, Australia.

^9^Department of Archaeology, Max Planck Institute for the Science of Human History, Kahlaische Straße 10, 07745, Jena, Germany.

^10^Extreme Events Research Group, Max Planck Institutes for Chemical Ecology, the Science of Human History, and Biogeochemistry, Jena, 07745, Germany.

*Corresponding author: [scerri@shh.mpg.de](mailto:scerri@shh.mpg.de).

**The Young MSA**

In West Africa, the argument for a young Middle Stone Age (MSA) has been made since at least the 1970s, typically on the basis of geomorphology and early radiocarbon dating (see [*21- 24*] for summaries). However, many of these claims were made are based upon interpretations and age estimates which would no longer be considered reliable. For example, at Asokrochona and Tema II in Ghana, arguments for an MIS 2 date were made on the basis of geological interpretation [*23*]. In Senegal, artefacts described as ‘Mousteroid’ and ‘Evolved Palaeolithic’ (featuring small foliates) were considered to be as young as 13.5 ka [*24*]. Early radiocarbon dates seemed to corroborate this narrative. In Cameroon, a possible *terminus* *ante quem* of 15 kyr uncal BP was obtained in the 1970s by radiocarbon from the MSA assemblage of Figuil-Louti, which features Levallois and discoidal cores and Levallois flakes [*65*]. At the MSA site of Bilma, in Niger, a *terminus ante quem* of 33.4 ± 2.5 kyr uncal BP was also obtained in the 1970s by radiocarbon, from an assemblage in stratified but secondary position [*25*]. Summaries of these dates led to the prediction that the MSA in West Africa should date to between 35 and 15 ka [*26*], as indeed OSL dating at Birimi in Ghana demonstrated [*26*].

More recently, work on various localities at Ounjougou and in the Lower Falémé valley have identified lithic assemblages from MIS 6 to MIS 2 which feature varied technologies within an MSA umbrella (e.g. [*22; 27-29*]). While these assemblages often feature relatively simple flake production – in part potentially attributable to the frequent use of low-quality raw materials such as sandstone, they feature consistent core reduction methods and retouched tool forms characteristic of the MSA. Core reduction methods include Levallois methods, and retouched forms include denticulates, sidescrapers and bifacially retouched points. The site of Tiemassas has revealed excavated MSA lithic assemblages with a consistent presence of alternate modes of Levallois flake production and discoidal reduction alongside low frequencies of retouched tools, predominately side scrapers, dated using OSL between 62-25 ka [*30-31*]. Finally, work in the Senegal Valley identified the late MSA (ca. 12 ka) site of Ndiayène Pendao, which features Levallois cores and flakes, denticulates, basally thinned pieces and core axes, with no trace of LSA characteristics, such as microliths, microblade cores, splintered pieces and segments [*33*]. Other undated sites in the Senegal Valley, such as Njideri and Madina Cheikh Omar, feature similar characteristics to Ndiayène Pendao [*32*].

**Laminia and Saxomununya site formation**

We interpret both Laminia and Saxomununya as cobble or gravel deposits that were subsequently exploited by humans to produce stone tools. The OSL age estimates for these deposits therefore provide direct ages for human activity at Laminia and a maximum age at Saxomununya. As well as their stratigraphic position at Laminia, this is indicated by generally low weathering and edge and arris sharpness. In both cases very few examples showed the kind of chipped edges we would expect from fluvial transport of artefacts. Even in the large sample of artefacts from Saxomununya, most of which are small and with the flakes having edges vulnerable to chipping, few examples show signs of damage that would indicate redeposition. By comparison, in a study of fluvial deposition in a small Welsh river, Hosfield and Chambers [*66*] found that lithics which moved just a few meters to a few tens of meters featured relatively frequent breakage (4-20 %) and, crucially, very common micro-flaking of the edges, with several scatters having more than two-thirds of the flakes featuring small chipping. It therefore seems highly unlikely that the assemblage has been transported and redeposited without being damaged.

Spatial aspects are also consistent with the hypothesis that the lithics at Laminia and Saxomununya were knapped on site, rather than being fluvially redeposited. At Laminia this relates to the exclusive presence of lithics in the upper part of the gravels, and their horizontally limited extent (we did not find lithic artefacts in nearby gravel bars). As well as their limited spatial distribution, the artefacts show no significant weathering or edge rounding suggesting that they have not been substantially impacted by fluvial activity. Likewise, at Saxomununya the high-density of lithics at a particular locality, in contrast to very low artefact frequencies elsewhere in the area, are parsimoniously interpreted as suggesting that Saxomununya was a raw material source rather that representing the fluvial deposition of artefacts.

Our interpretation of Saxomununya is that the artefacts have suffered only slight deflation and spread, and there is no evidence that they were extensively fluvially redeposited. If the Saxomununya lithics had been redeposited we would expect to see more of a biased size profile, whereas in reality the size structure seems consistent with on-site knapping. The average weight of cores is 21.5 grams, for flakes 7.7 grams, while that of chips and chunks is 2.2 grams. The distribution of weights by category is shown graphically in Fig. S9. We could alternatively point out that while many chips and chunks weigh less than 1 gram, the larger cores weigh over 100 grams. A similar point could be made in terms of metric dimensions. The simplest explanation of this is that the lack of evidence for size sorting reflects on site knapping. If the lithics had simply become part of the gravel load, we do not imagine it likely that lithics with such varied masses would be densely deposited in a single spot.

Many experiments have been conducted by archaeologists in order to explore how fluvial redeposition can be inferred and understood (e.g., [*66-75*]). This is a complex area of research, as many variables are involved (e.g. the size of the river, the nature of the geology). For instance, several studies have been conducted in the small Afon Ystwyth in Wales. Here the movement of lithics was tracked over a short distance at several times. While providing a useful baseline, the probable difference between such rivers and rivers such as the Falémé, which has a very high-flow during the wet season should be emphasized. Likewise, most studies have focused on larger lithics (e.g. handaxes, large flakes) than the small-sized lithics from Saxomununya. Nevertheless, from these various studies a body of knowledge exists on the behavior of lithic assemblages in fluvial settings. What tends to emerge is that small lithics are preferentially removed (e.g., [*67-69, 73, 75]*). Some studies that have not agreed with this interpretation concern the movement of lithics in small rivers over a few meters to a few tens of meters (e.g., [*66*]).

While our collection at Saxomununya was in the form of a thorough walk over, it was not possible to collect every single small chip and chunk (as would be achieving by sieving, for instance). Nevertheless, it does appear that the small fraction of lithics at the site is under-represented. We interpret this as indicating the removal of smaller lithics from the site (see also [*69-70*]). This is not surprising, given the presence of sandy sediments overlying the gravel rich layers which are preserved in the southern part of the site, but seem to have been eroded to the north. The site therefore indicates winnowing leading to a removal of part of the smallest size component, but the presence of multiple size classes in a dense scatter of fresh artefacts is parsimoniously interpreted as indicating on site knapping. In perhaps the most comparable study, Schick [*70*] studied the impact of fluvial activity to dozens of test assemblages in different settings in East Africa. This study found that most experimental scatters were rapidly and significantly altered. Scatters located in active channels were quickly destroyed beyond recognition and the lithics scattered downstream.

**Supplementary References**

1. Marliac, A. Introduction au Paléolithique du Cameroun septentrional. *L'Anthropologie* **91,** 521–557 (1987).
2. Hosfield, R.T. & Chambers, J. C. Flake modifications during fluvial transportation: Three cautionary tales. *Lithics* **24,** 57–65 (2003).
3. Isaac, G.L. Towards the interpretation of occupation debris: some experiments and observations. *Kroeber Anthropol. Papers* **37,** 31–57 (1967).
4. Schiffer, M.B. Towards the identification of formation processes. *Am. Antiquity* **48,** 675–706 (1983).
5. Petraglia, M.D. & Nash, D. T. The impact of fluvial processes on experimental sites. in *Natural formation processes and the archaeological record* (eds. Nash, D.T. & Petraglia, M.D.) 108–130 (Oxford BAR, 1987).
6. Schick, K. D. Experimentally-derived criteria for assessing hydrologic disturbance of archaeological sites in *Natural formation processes and the archaeological record* (eds. Nash, D.T. & Petraglia, M.D.) 86–107 (Oxford BAR, 1987).
7. Petraglia, M.D. & Potts, R. Water Flow and the Formation of Early Pleistocene Artifact Sites in Olduvai Gorge, Tanzania. *J. Anthropol. Arch.* **13,** 28–254 (1994).
8. Bertran, P. et al. Particle size distribution of lithic assemblages and taphonomy of Palaeolithic sites. *J. Archaeol. Sci.* **39,** 3148–3166 (2012).
9. Byers, D.A., Hargiss, E. & Finley, J.B. Flake Morphology, Fluvial Dynamics, and Debitage Transport Potential. *Geoarchaeology* **30,** 379–92 (2015).
10. Chu, W. *Fluvial processes in the Pleistocene of Northern Europe*. (BAR International Series 2016).
11. Chu, W. & Hosfield, R.T. Lithic artifact assemblage transport and microwear modification in a fluvial setting: A radio frequency identification tag experiment. *Geoarchaeology* **35,** 591–608 (2020).


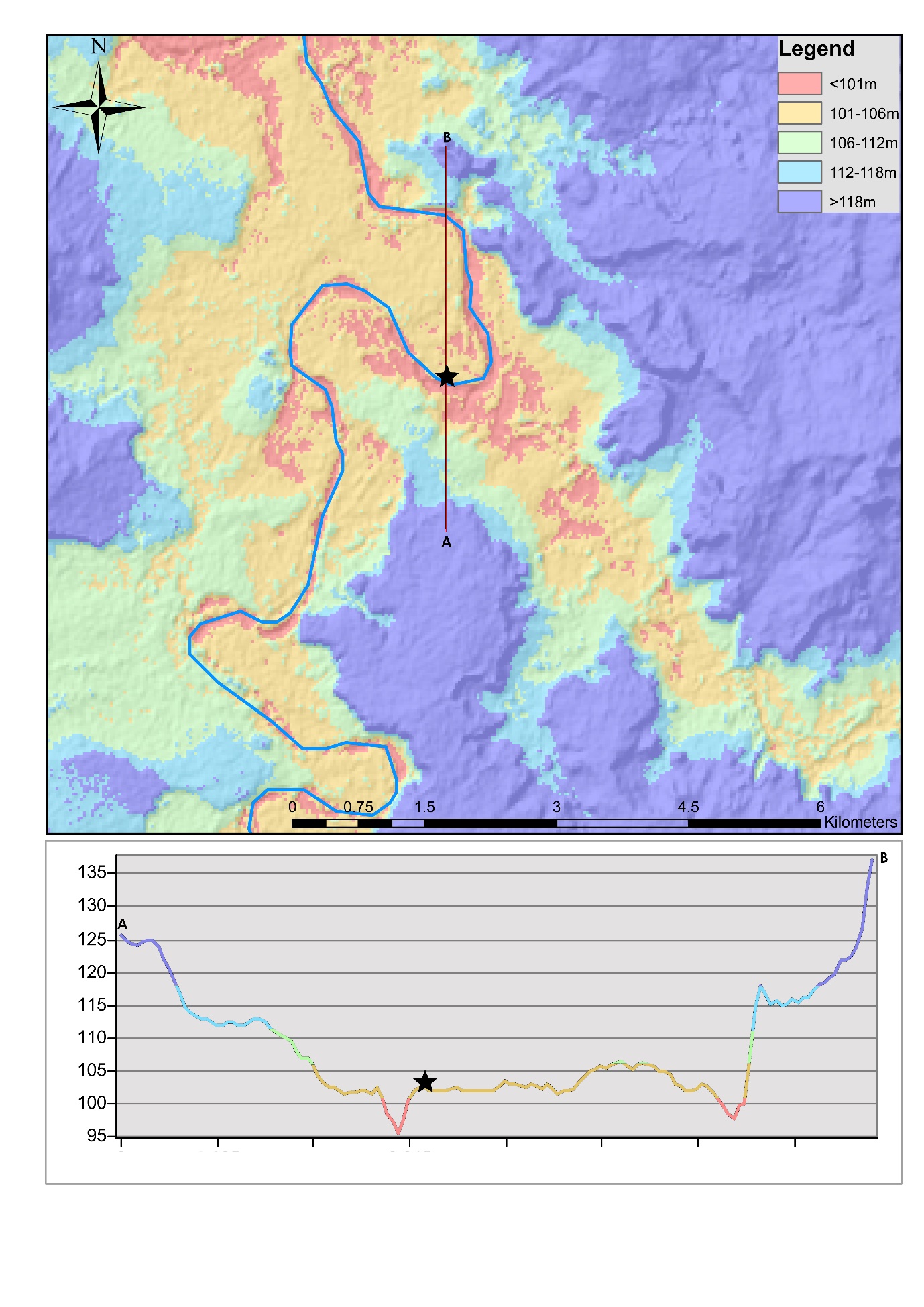


**Fig. S1:** (top) Digital terrain model (ALOS [JAXA]) illustrating the location of the archaeological site at Laminia (star) on a river terrace surface constrained in the immediate landscape to altitudes of 102-104m and a cross section across the Gambia Valley (red line); (bottom) elevation profile (generated from an ALOS DTM [JAXA] using ArcMap 10.5) across a section of the Gambia Valley (inidicated by the red line in top figure) illustrating the location of Laminia on a river terrace immediately adjacent to the modern, incised channel.


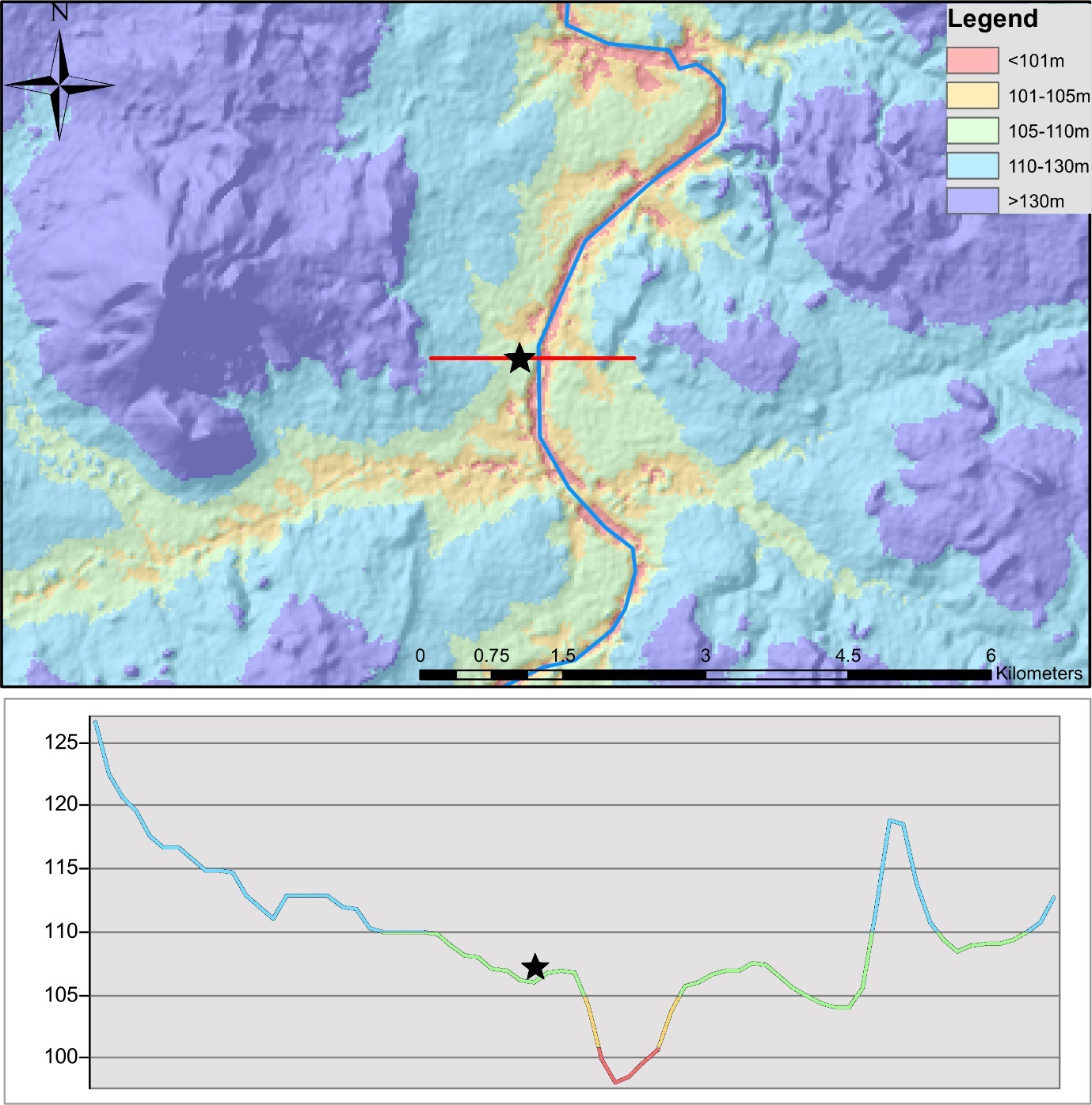


**Fig. S2:** (top) Digital terrain model (ALOS [JAXA]) illustrating the location of the archaeological site at Saxomununya (star) on a river terrace surface constrained in the immediate landscape to altitudes of 106-108m and a cross section across the Falémé Valley (red line); (bottom) elevation profile (generated from an ALOS DTM [JAXA] using ArcMap 10.5) across a section of the Falémé Valley (inidicated by the red line in top figure) illustrating the location of Saxomununya on a river terrace immediately adjacent to the modern, incised channel.


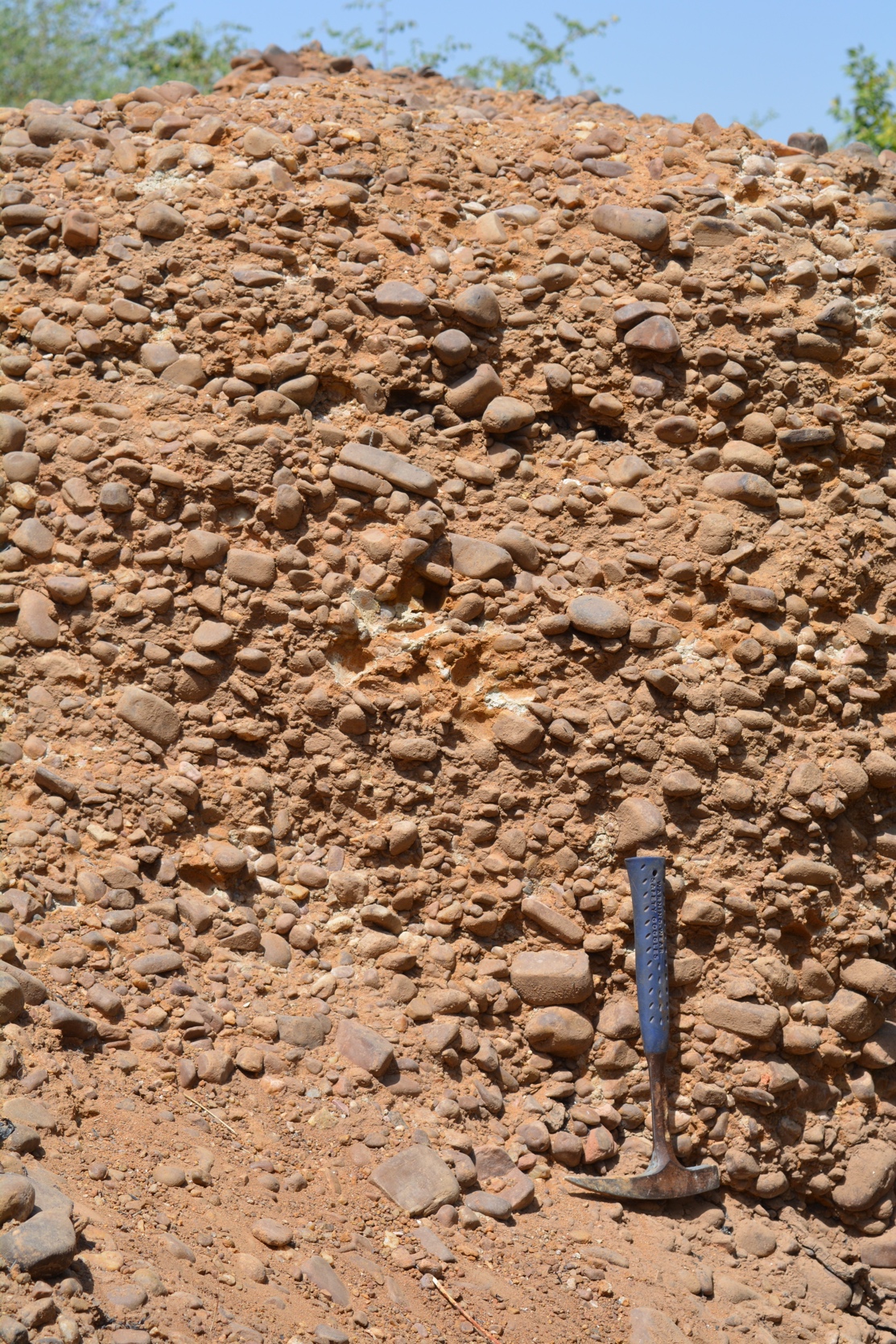


**Fig. S3.** Laminia section showing sediment type and gravel clasts.


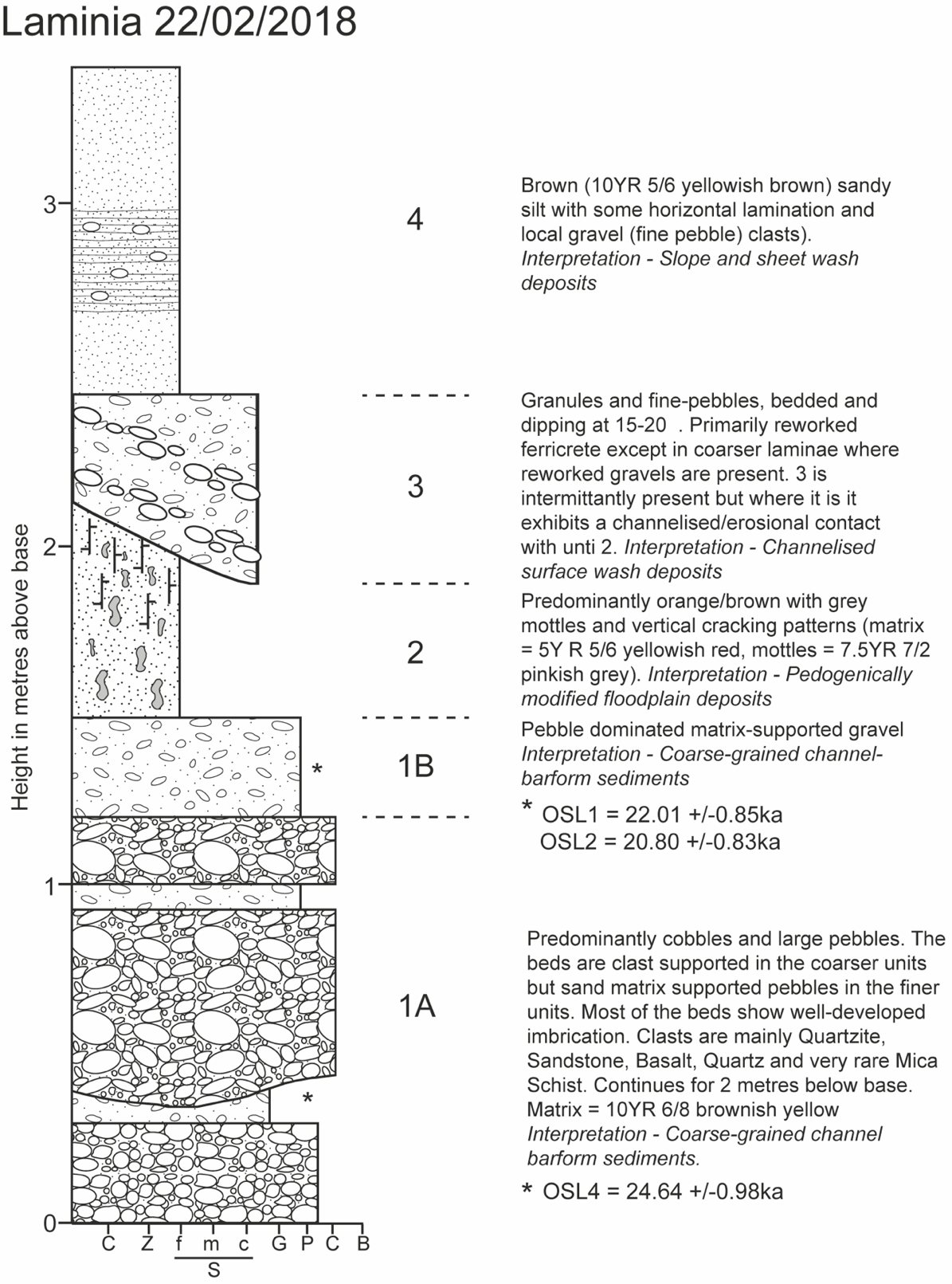


Fig. S4. Detailed sediment sequence from Laminia.


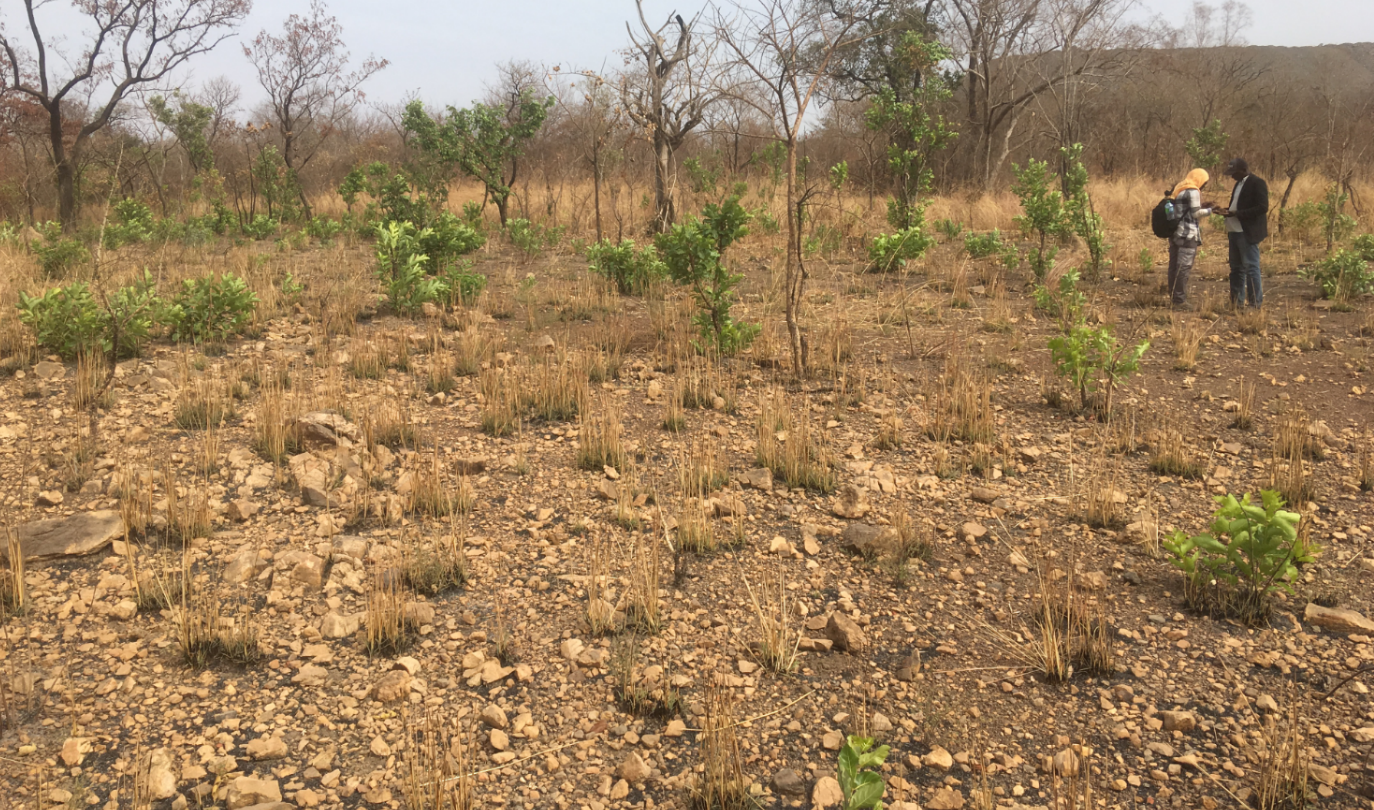


**Fig. S5.** Saxomununya**:** Surface of the site showing large numbers of lithics. The angular clasts are weathering from a bedrock exposure of very hard metamorphic rock. This slowed the waters of the ancestral Falémé River, leading to the deposition of a gravel deposit. This was then used by hominins as a raw material source. Lithics were found across the surface, and buried in sediments which have accumulated to the south (right on this image).


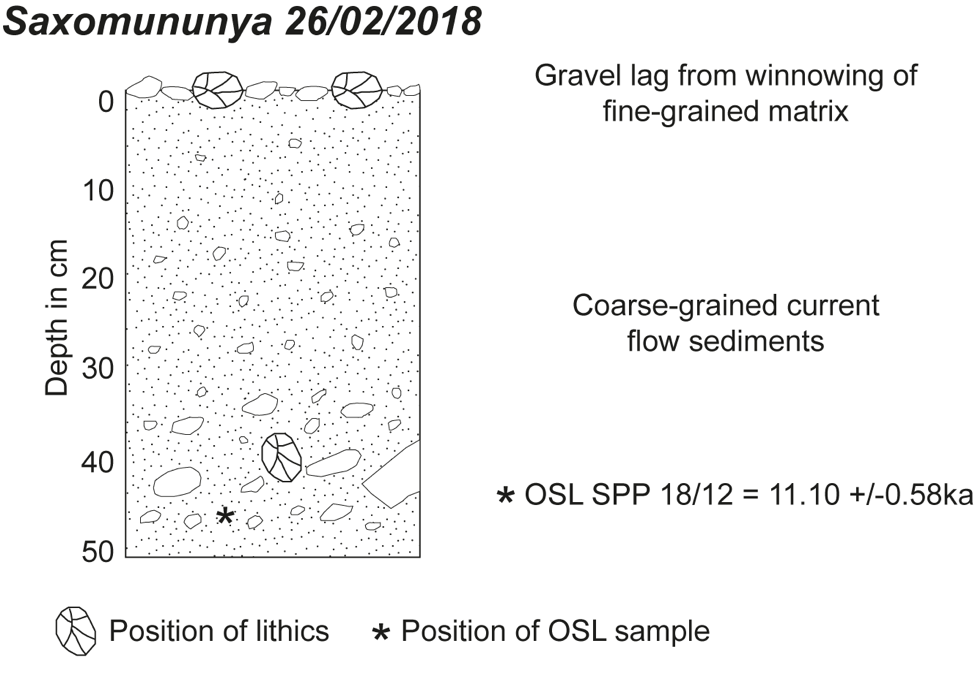


Fig. S6. Detailed sediment sequence from Saxomununya.


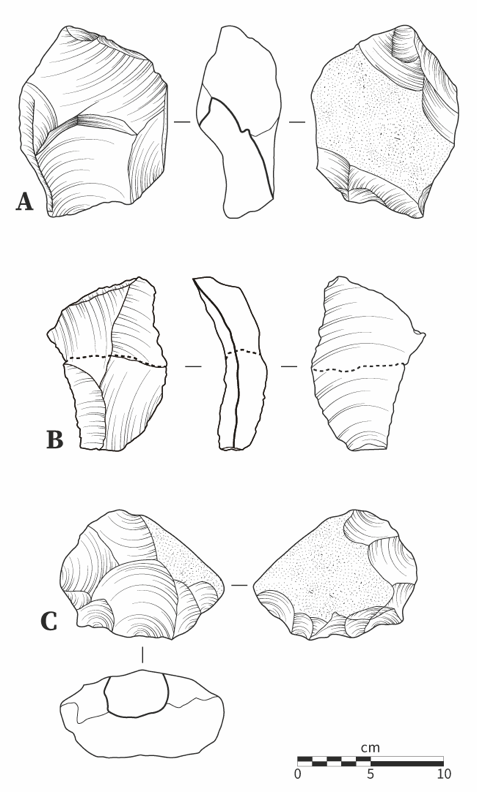


**Fig. S7.** Lithics from Laminia. A and C: Levallois Cores; B: Flake. Figure licensed under CC-BY-4.0.


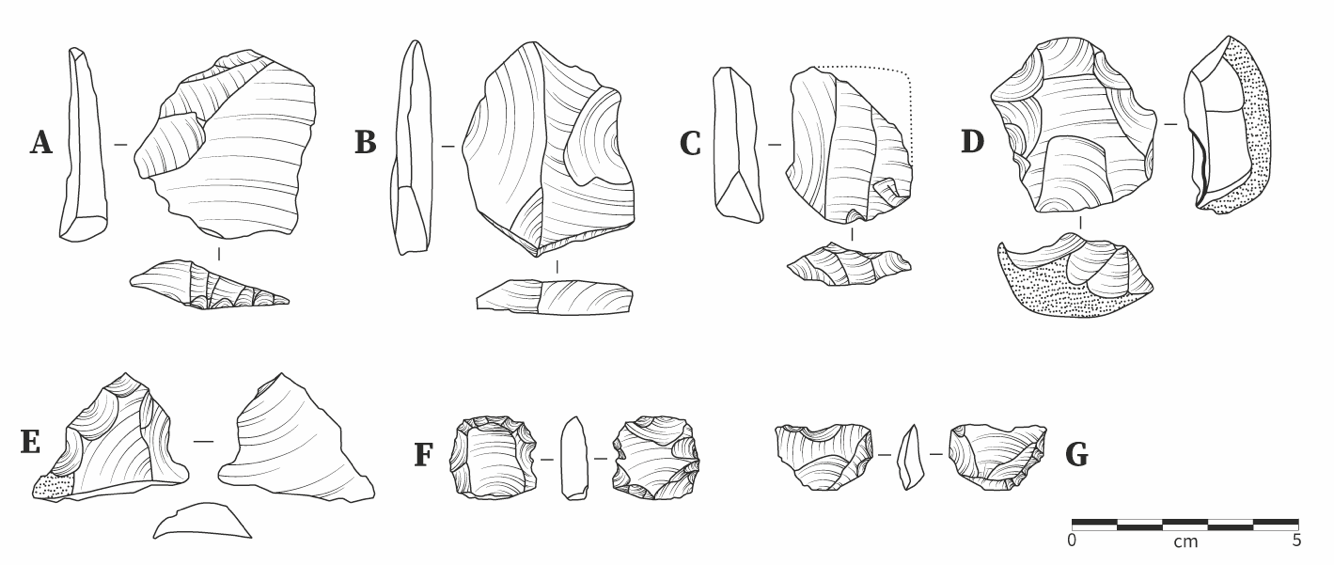


**Fig. S8.** Lithics from Saxomununya. A-C: Levallois flakes; D: Levallois Core; E: Broken retouched point tip; F-G: Side and end retouched pieces/scrapers. CC-BY-4.0.


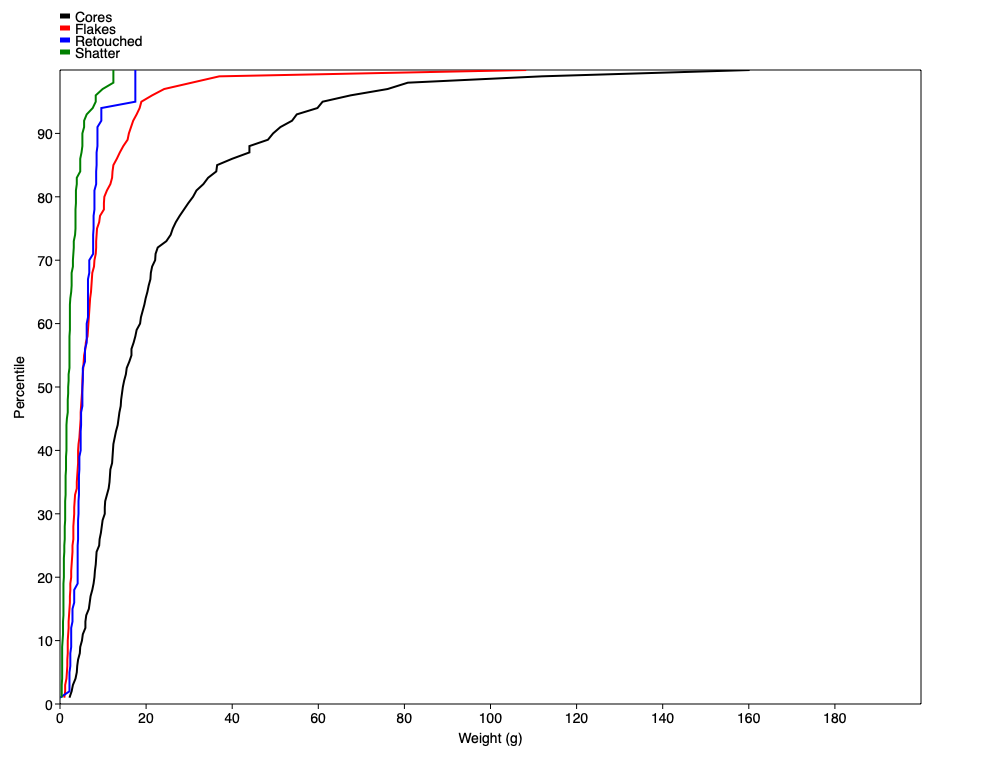


Fig. S9. Percentage size profile (grams) of Saxomununya lithics divided by categories.
